# Supplementary material for: Genetic Characteristics and Pathogenicity of a Novel Porcine Epidemic Diarrhea Virus with a Naturally Occurring Truncated ORF3 Gene
Source: Viruses. 2022 Feb 27;14(3):487. doi: 10.3390/v14030487 (PMC8955810; doi:10.3390/v14030487)
Supplement: Supplementary file 1 [file viruses-14-00487-s001.zip › Table S1.pdf]

Table S1. Oligonucleotide primers used for amplification by reverse transcription polymerase chain reaction.

Name	Sequence (5′-3′)	Nucleotide position	Product size(bp)
PEDV-M-F	CTCGGCTTGCATCACTCTT	25937	225
PEDV-M-R	GACCCAGTAGCAACCTTAT	26161	
PEDV-ORF3-F	GTCCTAGACTTCAACCTTACGAAG	24744	740
PEDV-ORF3-R	AACTACTAGACCATTATCATTCAC	25483	
1F	CGCCTACGGATAGTTAGCTCT	20	1601
1R	CAGCGATACTAAGAGTGGCAC	1620	
2F	CGTTGTGCCACTCTTAGT	1602	1622
2R	ATTGTGCCACCTTCCTCA	3223	
3F	CCAAACCACCAGCACTCAA	2857	1208
3R	CCCACGACCATAACCATCA	4064	
4F	GGAGGCAAGGATAGTGGTCAT	3996	1897
4R	GACATGGTGCCACTACAGGTT	5892	
5F	CTGGTCAGCCTAGTGATTCAG	5629	1895
5R	GTCCTCAGCAACAGCAGCATT	7523	
6F	CGTTGCAAGAGCACATTGGGT	7461	1668
6R	CCTACTAGCATACTGACGCAG	9128	
7F	GCCGCCAACTCTATCTCAA	9083	1924
7R	AACTCCGCTCACCACCAAT	11006	
8F	GCGACATGGCACATACACA	10549	1419
8R	GCTCGTCCACCCTCATTAT	11967	
9F	GCTGGTTGTACCTCTGTCT	11617	1709
9R	GGCAGGCAATTAGTCATAC	13325	
10F	GATAGGTGCTTGTGAGGAG	13020	1847
10R	GCACAACGCCATCATCAGA	14866	
11F	CGGTCTGATGATGGCGTTGTT	14855	1435
11R	CGTTACGTGCACCTGGATAGT	16289	
12F	GCATGAACACAAGCCACGTCT	15595	1837
12R	GTGACCCAACCAACCTCTAAC	17431	
13F	GTGCAGGCTAATGAGGGTT	17161	1771
13R	GGTGTAGGATCGTAATGGG	18931	
14F	GCGTTGTATGCCAAGCGTAAG	18887	1161
14R	GGGGCTAACGTAATCCACACT	18904	
15F	GATCATCTTGGTGCTGGCT	19933	766
15R	GTCGTGGTAGGCTAAGTGT	20698	
16F	GAAGGTAAGTTGCTAGTGCGTAA	20507	2351
16R	AGGTAGCCAATACTGCCAGATTT	22920	
17F	GTGGCCTGTGTTGGTGTATAG	22866	2122
17R	GGTGCCTCAAAGAAGACGCTT	24987	
18F	GGCGTCCTAGACTTCAACCTT	24740	849
18R	GGACTGCGCTATTACACAACC	25588	
19F	ACGGAGCTTCTTGATGGCA	25355	2105
19R	ACGTCAGACTTTGGCACAG	27460	
20F	CAAGTCTCGTAACCAGTCC	26975	1059
20R	CCCTCCATATCAACACCGT	28033	
